# An 11.8-fJ/Conversion-Step Noise Shaping SAR ADC with Embedded Passive Gain for Energy-Efficient IoT Sensors

**DOI:** 10.3390/s22030869

**Published:** 2022-01-24

**Authors:** Changhyung Choi, Jong-Wook Lee

**Affiliations:** Information and Communication System-on-Chip (SoC) Research Center, School of Electronics and Information, Kyung Hee University, Yongin 17104, Korea; choich92@khu.ac.kr

**Keywords:** analog-to-digital converter, successive approximation register, noise shaping, signal-to-noise, charge pump

## Abstract

Herein, we present a noise shaping successive-approximation-register (SAR) analog-to-digital converter (ADC) with an embedded passive gain multiplication technique. The noise shaping moves the in-band quantization noise from the signal band to out-of-band for improved signal-to-noise ratio (SNR). The proposed approach tackles the drawback of the previous active noise shaping (increased power and extra noise) and passive noise shaping (limited noise suppression and signal loss). Both noise shaping and gain multiplication are realized on-chip in an energy-efficient manner without an opamp. This approach uses only capacitors and switches in the finite impulse response (FIR) and infinite impulse response (IIR) filters. A comparator suppressing kickback noise is presented to handle the tradeoff between noise suppression and the filter capacitor size. The energy-efficient merged-capacitor switching (MCS) technique is effectively combined with rail-to-rail swing comparator and thermometer-coded capacitor array, which reduces the settling error in the digital to analog converter (DAC). The process-induced mismatch effect in the capacitive DAC is investigated using a behavioral model of the ADC. Additionally, we propose dynamic element matching (DEM) for the thermometer-coded capacitor array. The ADC is fabricated using a 0.18 μm CMOS process in an area of 0.26 mm^2^. Consuming 4.1 μW, the ADC achieves a signal-to-noise and distortion ratio (SNDR) of 66.5 dB and a spurious-free dynamic range (SFDR) of 79.1 dB. The figure-of-merit (FoM) of the ADC is 11.8 fJ/conversion-step.

## 1. Introduction

Demands for energy-efficient applications, such as the Internet of Things (IoT), battery-operated sensors, and wearable electronics, are continuously increasing. Ultra-low power consumption is required in these systems for signal sensing and processing to provide a long battery life. An analog-to-digital converter (ADC) is a key component in the processing of sensor output [1,2,3] and wireless communication [4,5]. Among various ADCs, successive approximation register (SAR) ADC is suitable for achieving high energy efficiency with low power consumption [6].

Typical SAR ADC consists of a digital-to-analog converter (DAC) realized using a capacitor array, a comparator, and SAR logic. The digital output for the analog input is obtained through charge redistribution in the capacitive DAC (CDAC). The SAR ADC provides medium resolution using very low power since the clocked comparator and capacitive switching consume only the dynamic power. One drawback of the SAR ADC is that the area of the CDAC needed to realize the binary weight increases rapidly with the resolution. When the number of CDAC bits is increased for high resolution, routing becomes more complicated in the SAR ADC. Additionally, the comparator’s input-referred noise and quantization noise limit ADC performance; designing high-resolution SAR ADC with low complexity is a challenging task.

The noise shaping technique has been actively investigated to address the challenge [7,8,9,10,11,12,13,14,15,16,17,18,19,20,21,22]. This technique moves the in-band quantization noise from the signal band to out-of-band for improved signal-to-noise ratio (SNR). The number of capacitors in the DAC can be reduced using noise shaping, simplifying the practical implementation of the SAR ADC. The previous work on the SAR ADC realizes the noise shaping filter using opamp and achieves a 10-bit effective number of bits (ENOB) using 8-bit CDAC [7]. The filter consists of finite impulse response (FIR) and infinite impulse response (IIR) filters. This approach shows that a relatively good noise shaping can be achieved even with a low-quality integrator for the IIR filter.

The residue remaining on the DAC after completing the digital conversion is the difference between the sampled input and a digital estimate. An opamp is used to process this small voltage [7,9,10]; the opamp consumes static power and introduces extra noise. A dynamic amplifier is used for the noise shaping filter to handle this issue [11,12]. A dynamic structure realizing the passive FIR and IIR filters can remove the static power consumption; however, the gain of the dynamic amplifier can be sensitive to supply voltage and temperature, and additional calibration may be needed [12]. Additionally, power consumption using this approach is still high, for example, 460 [11] and 84 μW [12]. Alternatively, a voltage–time–voltage converter can be used to achieve process-insensitive active residue processing [8]. Because there are two signal components, DAC output and filtered residue at the comparator input, the comparator with multi-input pairs is used [7,15,16,17]. To handle the small residue, the differential input pair for the residue is sized larger than the one other receiving the DAC output. This approach provides the advantage of the increased gain for processing the residue; however, the kickback noise of the comparator is proportionally increased with the size of the input pair (or the capacitance). Additionally, a multi-input comparator increases the input-referred noise.

The passive residue summation using a single input pair can be an alternative solution [18]; however, this approach achieves relatively weak suppression of the in-band quantization noise, and signal loss problems remain. In work [15], two capacitors added in the integration path increase the zero of the noise transfer function (NTF) to 0.75; however, the capacitor performing the residue sampling is reset after each conversion cycle, degrading the integration effect. Therefore, the previous approaches suffer from the tradeoff between gain, kickback, and input-referred noise. These results indicate that the noise shaping technique suitable for simple and power-efficient SAR ADC has not been fully investigated.

This paper proposes a simple and power-efficient noise shaping technique, which reduces the number of capacitors in the DAC. We embed a charge pump in the filter for passive gain multiplication to deal with the residue attenuation in the previous passive noise shaping. This approach uses only capacitors and switches in the FIR and IIR filters. Thus, noise shaping and gain multiplication are realized on-chip in an energy-efficient manner without an opamp. To handle the tradeoff between noise suppression and chip area, we propose a comparator canceling the kickback noise. The energy-efficient merged-capacitor switching (MCS) technique is effectively combined with the rail-to-rail comparator and the thermometer-coded capacitor array, which reduces the settling error in the DAC. The process-induced mismatch effect in the CDAC is investigated using a behavioral model of the ADC, and we propose a dynamic element matching (DEM) technique for the noise-shaping ADC. The proposed ADC fabricated in 180 nm CMOS demonstrates that the passive noise shaping technique enables ADC operation with an effective number of bits (ENOB) of 10.8-bit using a 9-bit CDAC. The measured result shows a significant improvement in the signal-to-noise and distortion ratio (SNDR) and spurious-free dynamic range (SFDR). Consuming 4.1 μW, the ADC achieves an SNDR of 66.5 dB and an SFDR of 79.1 dB with a figure-of-merit (FoM) of 11.8 fJ/conversion-step.

## 2. Design

### 2.1. ADC Operation

**Figure 1:**
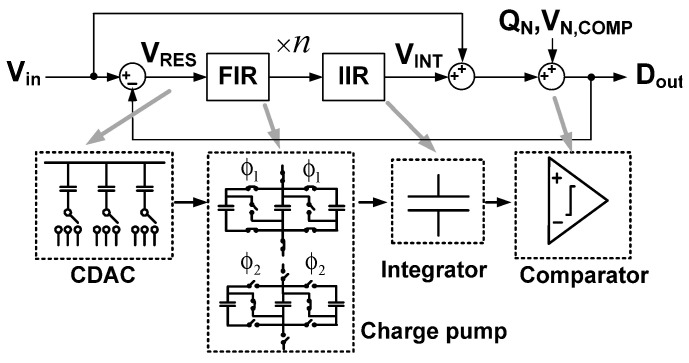
Functional representation of the proposed ADC.

Figure 1 shows the functional signal-flow diagram of the proposed ADC. After the sampling and conversion, the residue *V*_RES_, which is the difference between the analog input *V*_in_ and the digital estimate *D*_out_, remains on the top plate of the CDAC. *V*_RES_ is integrated by the FIR and IIR filters. The ADC feedforwards *V*_in_ to the quantizer, and the integrated residue *V*_INT_ is added with the *V*_in_ to generate *D*_out_ [9]. Considering the quantization noise *Q*_N_ and the comparator noise *V*_N,COMP_, *D*_out_ can be expressed as

(1) $$D_{\mathrm{out}}=V_{\mathrm{in}}+\frac{1}{1+L(z)}(Q_{N}+V_{N,\mathrm{COMP}})$$

where *L*(z) = *V*_INT_(z)/*V*_RES_(z) is the filter transfer function. Using the proper NTF = 1/[1+ *L*(z)], both *Q*_N_ and *V*_N,COMP_ can be reduced at the expense of bandwidth. Because *V*_RES_ is less than one least significant bit (LSB), proper processing of *V*_RES_ is important to achieve noise shaping. In this work, *V*_RES_ is boosted by passive multiplication inside the FIR filter. The multiplication is realized using the capacitive charge pumping. Switches are controlled to sample *V*_RES_ in parallel, and the connection is changed to series to achieve the multiplication of *n*, which is the number of FIR capacitors. The IIR filter is realized using a single capacitor for integrating the output of the FIR filter.

**Figure 2:**
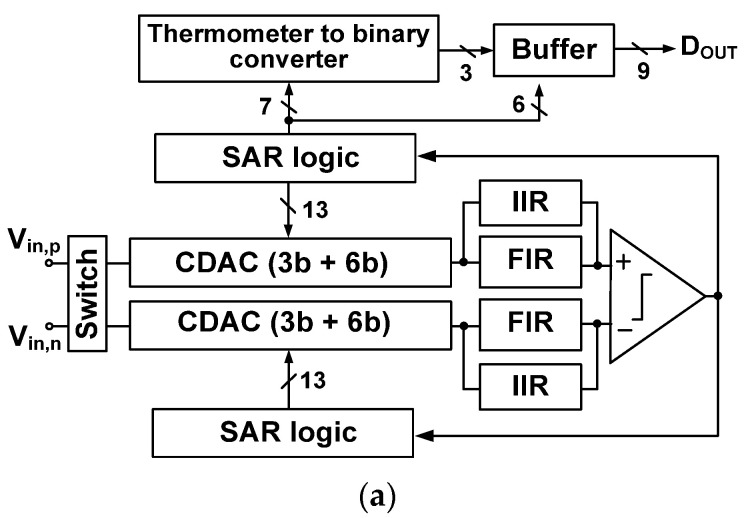

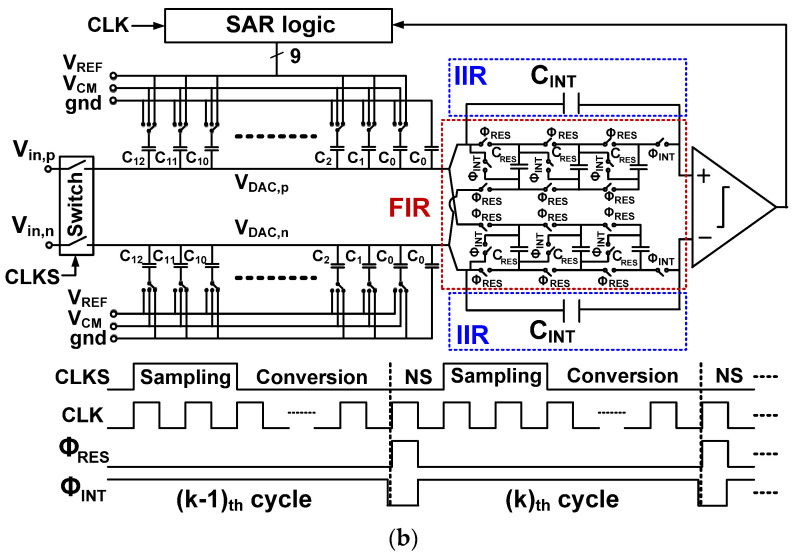
(**a**) Block diagram of the proposed ADC; (**b**) schematic of the proposed noise-shaping SAR ADC with timing waveforms. The *V*_REF_ = 1.8 V is the reference voltage.

Figure 2a shows the block diagram of the proposed ADC. Top-plate sampling is performed using a bootstrapped switch [23]. The MCS technique is used for the DAC, chosen for its high energy efficiency and constant common-mode (CM) operation [24]. To realize noise shaping, the FIR and IIR filters are located between the CDAC and the comparator. The integrated residue is handled using the residue-summation technique [18], which allows processing the residue using the comparator having a single input pair.

Figure 2b shows the schematic of the proposed ADC with the related timing waveforms. The *V*_DAC,p_ and *V*_DAC,n_ are the top plate voltages of the positive and negative DAC, respectively. The settling error in the DAC can be reduced using the thermometer coding, which is used for the upper 3-bit. Binary coding is used for the remaining 6-bit; the DAC consists of seven thermometer-coded capacitor array *C_i_* (*i* = 6 to 12) and six binary-weighted array *C_j_* (*j* = 0 to 5). We note that the seven thermometer-coded elements represent a 3-bit binary code. Therefore, overall DAC consists of a 9-bit. When the comparator determines the LSB, the result of the last decision (ninth decision) is not fed back to the DAC. This operation explains why the 9-bit DAC generates the digital output having 8-bit accuracy. Additionally, a residue remains at the top plate of the DAC, which is the difference between the sampled input and an 8-bit digital estimate [7]. Each FIR filter consists of residue sampling capacitors *C*_RES_. Each IIR filter consists of an integrating capacitor *C*_INT_. The sampling clock CLKS is used for the bootstrapped switch, and the ADC operates synchronously with the clock signal CLK. After sampling and conversion operations are performed, the noise shaping (NS) cycle follows. Residue processing is performed using the two-phase signals, Φ_RES_ for residue sampling and Φ_INT_ for residue integration.

### 2.2. Noise Shaping Operation

**Figure 3:**
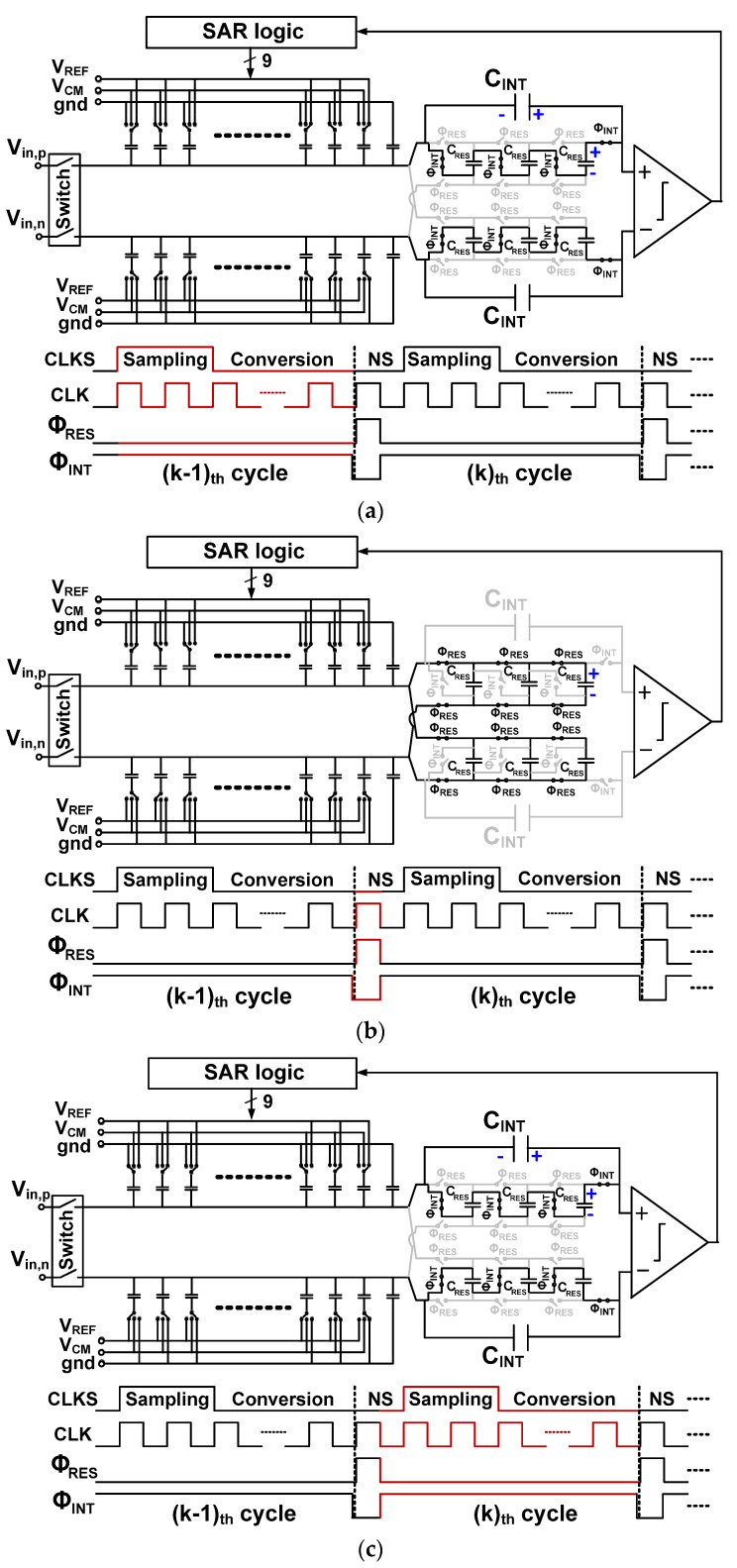
Operation of the noise shaping ADC. (**a**) (*k* − 1)_th_ cycle for sampling and digital conversion, (**b**) (*k* − 1)_th_ cycle for residue capture, and (**c**) *k*_th_ cycle for charge pumping and residue integration.

Figure 3a shows the sampling and conversion operations in the (*k* − 1)_th_ cycle. During conversion, *V*_DAC,p_ and *V*_DAC,n_ change around the CM voltage *V*_CM_. After the digital conversion, there are residue voltages, *V*_RES,p_ and *V*_RES,n_, on the positive and negative DAC, respectively, which is the difference between the sampled input and an 8-bit digital estimate. The previous conversion cycle sets the voltage *V*_INT_[*k* − 1] across *C*_INT_. Switches are controlled to connect *C*_RES_ in series, which is in parallel with *C*_INT_. Then, the voltage *V*_CRES_[*k* − 1] across each *C*_RES_ is *V*_INT_[*k* − 1]/*n*. Here, *n* is the number of *C*_RES_, and the case of *n* = 3 is shown.

Figure 3b shows the operation during the NS cycle when Φ_RES_ is high (Φ_INT_ is low). During this time, the residue is captured. The residue 2*V*_RES_ = (*V*_RES,p_ − *V*_RES,n_) on the top plate of the differential DAC is sampled on *C*_RES_. At this time, six *C*_RES_ are connected in parallel with the DAC. The *V*_RES_ is transferred from the DAC to *C*_RES_ by charge redistribution. Therefore, *V*_RES_ is scaled by a factor *α*, which is the ratio of *C*_DAC_ and *nC*_RES_ as

(2) $$\alpha =\frac{C_{\mathrm{DAC}}}{C_{\mathrm{DAC}}+nC_{\mathrm{RES}}}$$

where *C*_DAC_ is the sum of the DAC capacitors. To obtain *V*_CRES_, we need to consider another charge from *C*_INT_. In the previous cycle, we have *V*_CRES_[*k* − 1] = *V*_INT_[*k* − 1]/*n*. Considering that the charge from *C*_INT_ is shared between *nC*_RES_ and *C*_DAC_, we can express *V*_CRES_ as

(3) $$V_{\mathrm{CRES}}[k]=\frac{C_{\mathrm{DAC}}}{C_{\mathrm{DAC}}+nC_{\mathrm{RES}}}\cdot 2V_{\mathrm{RES}}[k-1]\;+\frac{nC_{\mathrm{RES}}}{C_{\mathrm{DAC}}+nC_{\mathrm{RES}}}\cdot \frac{V_{\mathrm{INT}}[k-1]}{n}=2\alpha \cdot V_{\mathrm{RES}}[k-1]\;+(1-\alpha )\cdot \frac{V_{\mathrm{INT}}[k-1]}{n}$$

The first term considers the charge transferred from *C*_DAC_ to *C*_RES_. The second term accounts for the charge sharing between *nC*_RES_ and *C*_DAC_, occurring when the charge stored in *C*_INT_ is transferred to *nC*_RES_.

Figure 3c shows the operation during the NS cycle when Φ_RES_ is low (Φ_INT_ is high). During this time, both voltage multiplication and residue integration are performed. After the residue capture, switches are controlled to connect *nC*_RES_ in series. Then, *V*_CRES_ is charge pumped and multiplied by *n*. The boosted voltage is scaled by the factor *β*, which accounts for the charge sharing between *n* series-connected *C*_RES_ and *C*_INT_ as

(4) $$\beta =\frac{(1/n)C_{\mathrm{RES}}}{C_{\mathrm{INT}}+(1/n)C_{\mathrm{RES}}}.$$

The integration with a gain of *β* is performed during the high Φ_INT_ cycle. By adding the value *V*_INT_[*k* − 1] of the previous cycle, which is charge shared between *C*_INT_ and *C*_RES_/*n*, we can express *V*_INT_[*k*] of the *k*th cycle as

(5) $$V_{\mathrm{INT}}[k]=\frac{C_{\mathrm{INT}}}{C_{\mathrm{INT}}+(1/n)C_{\mathrm{RES}}}\cdot V_{\mathrm{INT}}[k-1]\;+\frac{(1/n)C_{\mathrm{RES}}}{C_{\mathrm{INT}}+(1/n)C_{\mathrm{RES}}}\cdot nV_{\mathrm{CRES}}[k]=(1-\beta )\cdot V_{\mathrm{INT}}[k-1]+\beta \cdot nV_{\mathrm{CRES}}[k].$$

Using (3) and (5), we obtain

(6) $$V_{\mathrm{INT}}[k]=(1-\alpha \beta )V_{\mathrm{INT}}[k-1]\;+2n(\alpha \beta )V_{\mathrm{RES}}[k-1]\;.$$

The *L*(z) is obtained by rearranging (6) as

(7) $$L(z)=\frac{2n\alpha \beta z^{-1}\;}{1-(1-\alpha \beta )z^{-1}}.$$

After the integration is finished during the NS cycle, the next *k*th cycle for the sampling and conversion starts. At this time, the integrated residue is added to the CDAC at the comparator input.

**Figure 4:**
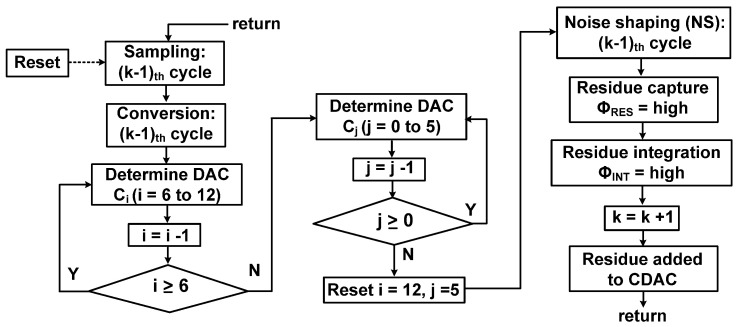
Flowchart of the proposed ADC operation during (*k* − 1)_th_ cycle.

Figure 4 shows the flowchart of the proposed ADC operation. After sampling the analog input, the DAC is determined by the binary search algorithm. Using the comparator output, the DAC switch is connected to either *V*_REF_ or gnd, repeated seven times for the thermometer-coded capacitor array *C_i_* (*i* = 6 to 12) and six times for the binary-weighted array *C_j_* (*j* = 0 to 5). Then, the noise shaping cycle follows, consisting of one cycle for residue capture (Φ_RES_ = high) and another cycle for residue integration (Φ_INT_ = high). After the NS cycle, the integrated residue is added to the CDAC at the comparator input during the next *k*_th_ cycle for the sampling and conversion.

### 2.3. Analysis of Noise Suppression

Using (7), we obtain the NTF as

(8) $$\mathrm{NTF}=\frac{1-(1-\alpha \beta )z^{-1}}{1+[(2n+1)\alpha \beta -1]z^{-1}}.$$

**Figure 5:**
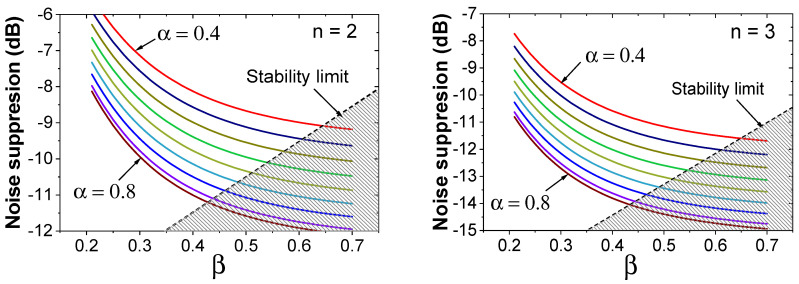
Noise suppression as a function of *β* for various α values.

**Figure 6:**
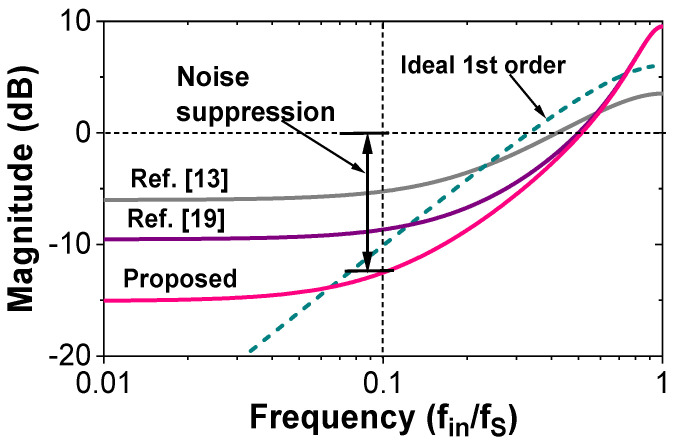
Calculated NTF as a function of normalized frequency.

Using the magnitude of NTF, we obtain the in-band quantization noise reduced by noise shaping. Considering the tradeoff between the chip area and the passive gain, we investigate the two cases of *n* = 2 and *n* = 3. Figure 5 shows the noise suppression calculated at (*f*_S_/*f*_in_) = 0.1 (See Figure 6). Here, *f*_S_ is the sampling frequency of CLK, and *f*_in_ is the input frequency. The result shows the improved noise suppression (2–3 dB) achieved using *n* = 3.

The noise suppression increases with *α* and *β* values; however, it saturates with increased values. When we consider the residual kickback from a clocked comparator, the size of *C*_RES_ cannot be reduced (increased *α*) to an arbitrarily small value. For the given *C*_DAC_, the kickback effect on *V*_DAC_ increases with *α*. Additionally, the stability condition (the pole of NTF should be inside the unit circle in the z-domain) sets the upper limit for *α* and *β* values. Because *C*_RES_ is fixed by the selected *α* value, *C*_INT_ is reduced with increasing *β*. When *C*_INT_ is reduced, the kickback noise increases. Additionally, *C*_INT_ should be sized considering the kT/C noise [7] and the charge sharing with the *C*_DAC_. Because there is no external charge supplied into the passive filter, the tradeoff is inherent in the ADC based on passive noise shaping. Using circuit simulations, we investigate the kickback noise and choose *n* = 3, *α* = 0.7, and *β* = 0.3 so that the noise is less than 0.5 LSB. Noise suppression up to 15 dB is achieved at low *f*_in_ using these parameters.

Table 1 shows the various NTF expression and the calculated noise suppression. Figure 6 shows the comparison of the magnitude of NTF. The result shows that our approach achieves 7.23 dB and 3.81 dB better noise suppression than the previous works [13,18], respectively.

**Figure 7:**
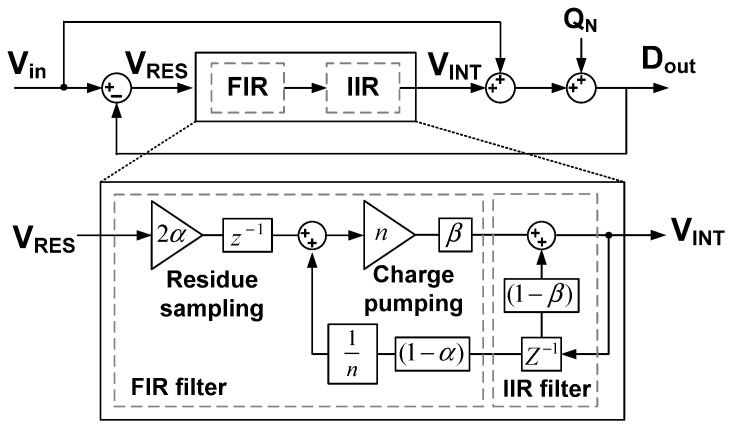
Behavioral model of the proposed ADC.

**Figure 8:**
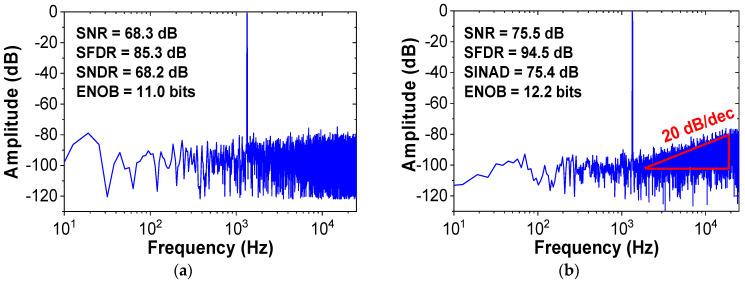
Output spectra of the ADC (**a**) without noise shaping and (**b**) with noise shaping for the CDAC mismatch rate of 0.5%. *f*_in_ = 1.33 kHz and *f*_S_ = 52 kHz.

Using (6), we implement the behavioral model of the noise shaping ADC, as shown in Figure 7. The charge pump is modeled using an amplifier with a gain of *n*. Comparator and kT/C noise are not considered as they experience the same NTF as the quantization noise [7]. Effect of process variations in the CDAC can be considered by including random mismatch rate. Simulations are performed to investigate the performance improvement by the proposed noise shaping technique. Figure 8 shows the output spectrum of the proposed ADC obtained from the fast Fourier transform (FFT) spectrum with 8192 points. The result confirms the first-order noise shaping achieved by the proposed method. When noise shaping is enabled, the SNR and SNDR increase by 7.2 and 9.2 dB, respectively.

**Figure 9:**
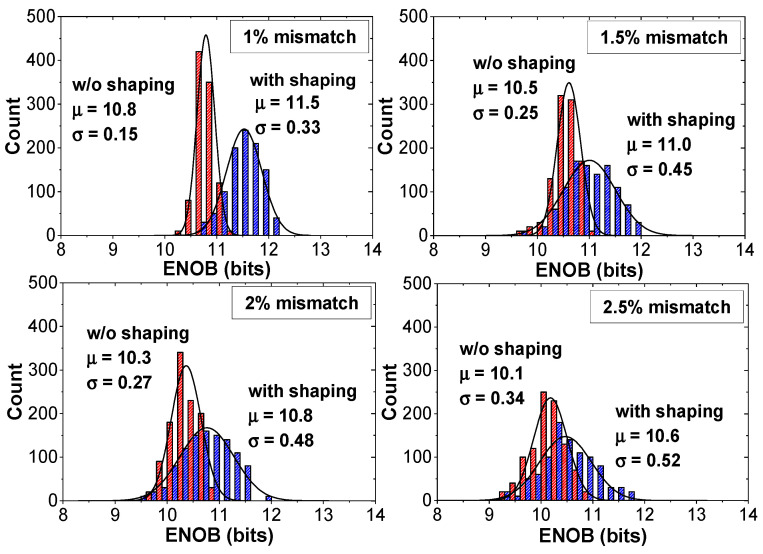
Probability distributions of the ENOB for different CDAC mismatch rate.

**Figure 10:**
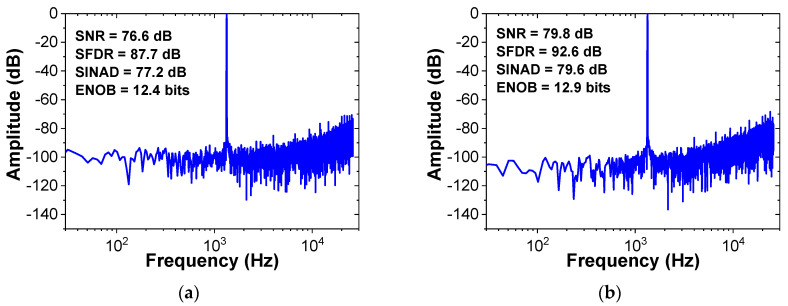
Output spectra of the ADC. (**a**) Previous work and (**b**) proposed work. *f*_in_ = 1.33 kHz, OSR = 10.

The performance improvement by noise shaping can be affected by the CDAC mismatch. We investigate the random mismatch effect in the CDAC using the behavioral ADC model. Figure 9 shows the probability distributions of the ENOB for different CDAC mismatch rates obtained by 1000 Monte Carlo simulations. When the mismatch increases from 1% to 2%, the average ENOB decreases from 11.5 to 10.8 bits. Considering the mismatch effect, we determine the unit capacitor size (*C*_0_ = 21 fF) to keep the mismatch less than 1%. The linearity characteristics affected by the CDAC mismatch can be further improved using foreground calibration [17]. Figure 10a shows the output spectrum of the previous work [18], which uses a 13-bit DAC (10-bit CDAC, 2-bit for redundancy, and 1-bit for noise shaping). Because of additional capacitor switching for noise shaping, three extra cycles are needed for A/D conversion. The results are obtained from the FFT spectrum with 4096 points. Figure 10b shows the output spectrum of the proposed work, which uses a 9-bit CDAC and a passive filter. Our work uses only one additional clock for A/D conversion. Compared to the previous work [18], our work achieves increased zero value in the NTF. The results show that our work using 1-bit smaller DAC achieves increased SNR and SNDR by 3.2 and 4.9 dB, respectively.

### 2.4. Comparator for Reduced Kickback

**Figure 11:**
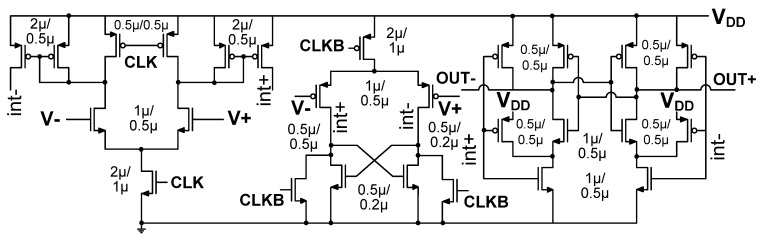
Schematic of the comparator having complementary differential input pairs. *V*_DD_ = 1.8 V.

The previous work uses cascoding transistors to reduce the kickback noise [25]. Because the comparator is designed for the monotonic switching algorithm for the SAR ADC, it is implemented with a PMOS differential input pair. When the MCS algorithm is used, the *V*_CM_ of the DAC is fixed during the conversion. When the previous comparator is used for MCS, it can result in a relatively large offset at the input of the comparator, especially during LSB conversion. Figure 11 shows the schematic of the comparator used in this work. The cascoding transistors are removed, and complementary differential input pairs are used, allowing rail-to-rail input range. We note that the comparator does not have a separate input pair for the residue. The proposed comparator uses two clock signals, CLK and CLKB. Consider the *V*_DAC,n_ on the negative branch DAC, connected to the negative terminal *V*- of the input pair. The CLK and CLKB signals generate two kickback noise components. Because CLKB is an inverted signal of CLK, the kickback noise in *V*_DAC,n_(CLKB) is the inverted version of the noise in *V*_DAC,p_(CLK). Because the complementary input pair generates the two kickback noise in opposite directions, they can be canceled out. Similarly, the kickback noise on *V*_DAC,p_ connected to the positive terminal *V*+ of the input pair is canceled. The residual kickback noise depends on capacitance matching between the two signal paths.

## 3. Measured Results

**Figure 12:**
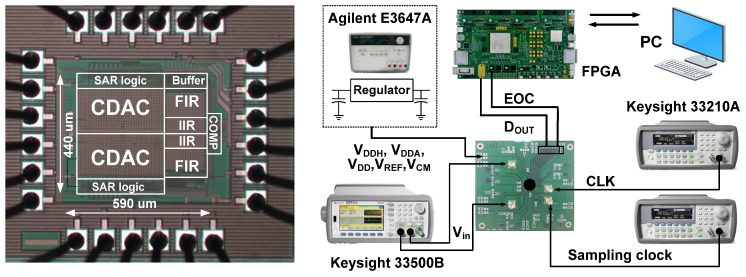
Microphotograph of the fabricated ADC. Measurement setup is also shown.

Figure 12 shows the microphotograph of the ADC fabricated in the 0.18 μm CMOS process. The core area is 0.26 mm^2^. The overall power consumption is 4.1 μW, including 1.2 μW for the reference buffers. Analog, digital, and SAR logic consume 82.4%, 9.3%, and 8.3%, respectively. The measurement setup is also shown. The power supplies for the analog and digital blocks of the ADC are separated. They are stabilized using 1000 μF bypass capacitors and low-dropout (LDO) regulators. A field-programmable gate array (FPGA) board collects the ADC output.

**Figure 13:**
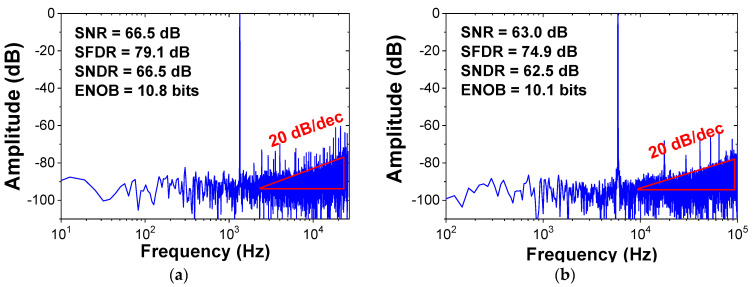
Measured output spectrum of the ADC. (**a**) *f*_in_ = 1.33 kHz and *f*_S_ = 52 kS/s and (**b**) *f*_in_ = 8 kHz and *f*_S_ = 180 kS/s.

**Figure 14:**
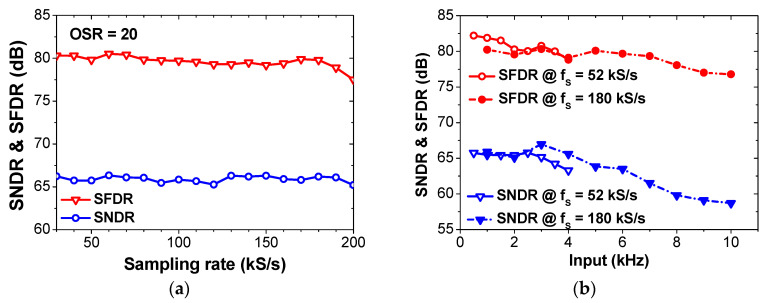
(**a**) Measured SNDR and SFDR as a function of the sampling rate. (**b**) Measured SNDR and SFDR as a function of the input frequency.

**Figure 15:**
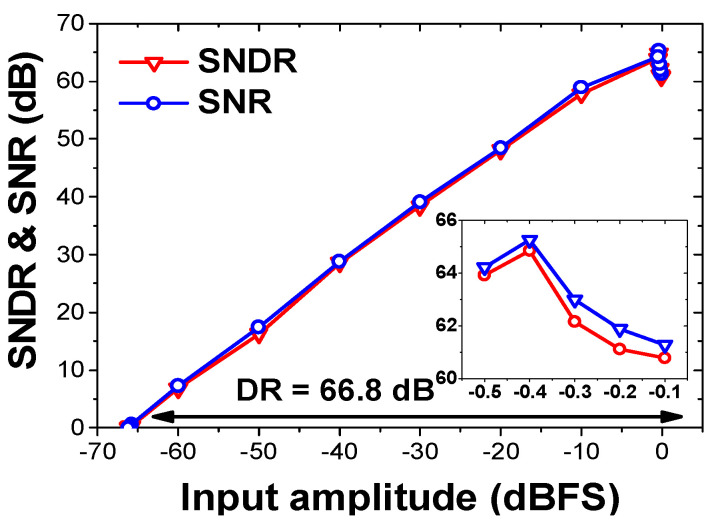
Measured dynamic range.

**Figure 16:**
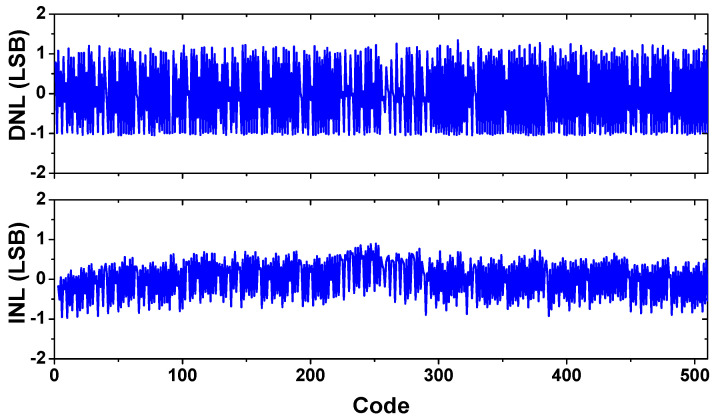
Measured static performance of the ADC.

Figure 13a shows the measured output spectrum using *f*_in_ = 1.33 kHz and *f*_S_ = 52 kS/s. The result is obtained from the FFT spectrum with 8192 points. The peak SNDR, SFDR, and ENOB are 66.5, 79.1, and 10.8 bits, respectively. Figure 13b shows the measured output spectrum at increased *f*_in_ = 8 kHz and *f*_S_ = 180 kS/s. Figure 14a shows the measured SNDR and SFDR as a function of *f*_S_. The result shows that the dynamic ADC performance is relatively constant, up to 180 kS/s. Figure 14b shows the measured SNDR and SFDR as a function of *f*_in_ for two sampling rates. The result shows that the dynamic performance gradually increases with the oversampling ratio (OSR). Figure 15 shows the measured dynamic range at *f*_in_ = 1.33 kHz and *f*_S_ = 52 kHz. Peak SNR and SNDR are measured with an input amplitude of −0.4 dBFS. Figure 16 shows the static linearity of the ADC. The result is obtained using a histogram test of 260,000 samples. The peak differential non-linearity (DNL) is +1.34/−1.05 LSB, and the peak integral non-linearity (INL) is +0.89/−0.96 LSB. Because the capacitors in the IIR and FIR filter are dynamically reconfigured, the exact binary weight condition cannot be satisfied for the CDAC. The result indicates the tradeoff in the design of the noise shaping ADC; the static performance is traded for improved dynamic performance.

The mismatch in the CDAC can affect the ADC linearity, and the DEM technique can be used to address the issue [9,10,19]. Either random or cyclic selection can realize the DEM. The cyclic selection uses the output of each conversion determined by the cumulated sum of the elements that are cyclically selected [26]. Two building blocks are usually used [27]. The first is the pointer that indicates the unit element used as the starting point for the DAC operation. The second is a decoder that maps the relationship between the thermometer-code and DAC unit elements. The pointer can be realized using an accumulator and a register. To reduce the implementation complexity, we use a binary counter to implement the pointer. Because the mismatch effect increases with the capacitor size, the DEM is used for the thermometer-coded capacitor array [23]. The binary-weighted arrays are not used for DEM; this approach requires sufficient intrinsic linearity for binary-weighted capacitors.

**Figure 17:**
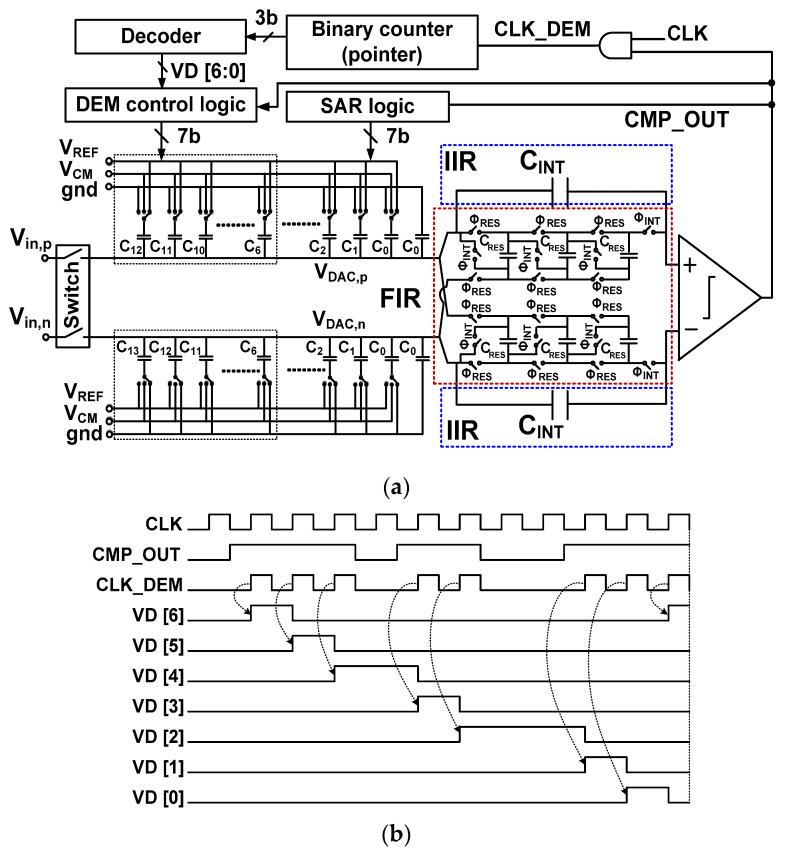
(**a**) Block diagram of the noise shaping ADC with the DEM. (**b**) Timing waveform of the DEM logic.

Figure 17a shows the block diagram of the noise-shaping ADC with the DEM logic. The thermometer-coded capacitor arrays are controlled using the output VD [6:0] of the 3 to 7 decoder. A binary counter, clocked by the comparator output CMP_OUT, is used as a pointer that determines the unit capacitor in the DAC. When CMP_OUT becomes high, the pointer is increased. The decoder receives the 3-bit output from the counter and decides the connection sequence of the thermometer-coded capacitors. The DEM is enabled only for seven clocks after input sampling. For this reason, we use a separate DEM control logic instead of the SAR logic. Figure 17b shows the related timing waveform. The CLK_DEM is enabled when CMP_OUT becomes high, increasing the pointer. The rising edge of the decoder output VD [6:0] triggers the DEM control logic to switch the bottom plate of the capacitors.

**Figure 18:**
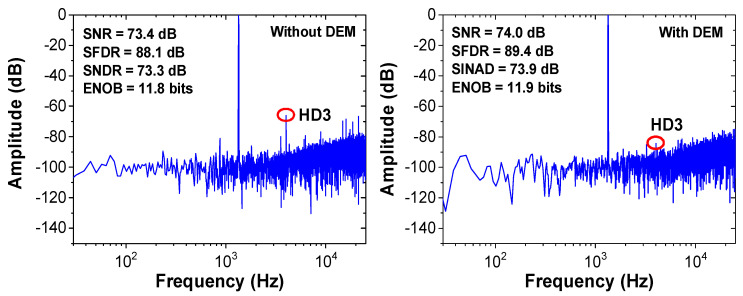
Comparison of the dynamic performance with and without the DEM.

**Figure 19:**
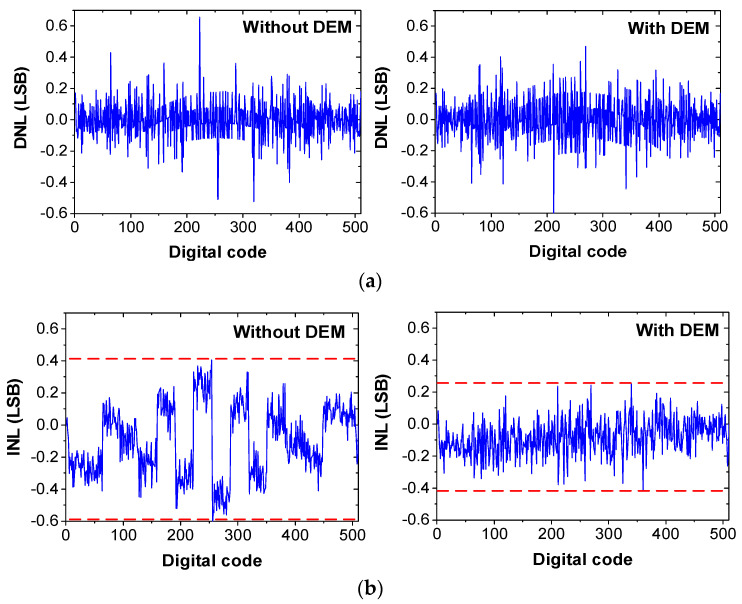
Comparison of the static performance of the ADC with and without the DEM. (**a**) DNL, (**b**) INL.

We implement the behavioral model of the noise-shaping ADC with the DEM logic. Figure 18 shows the dynamic performance of the ADC with and without DEM, obtained using a 1% CDAC mismatch. Without the DEM, the third harmonic level is located at around −67 dB, which is reduced to −84 dB using the DEM. Figure 19 shows the static performance with and without DEM. A total of 260,000 samples are used. The peak DNL is +0.66/−0.61 LSB, and the peak INL is +0.4/−0.61 LSB without the DEM. Using the DEM, peak DNL is reduced to +0.47/−0.62 LSB, and the peak INL is reduced to +0.25/−0.42 LSB. The results show that the linearity of the noise-shaping ADC can be improved using the DEM.

Table 2 shows the comparison with the previous works. Schreier’s figure-of-merit (FOMs) is defined as

(9) $${\mathrm{FOM}}_{S}=\mathrm{SNDR}+10\log_{10}(\mathrm{BW}/\mathrm{Power})\;[\mathrm{dB}]$$

where the bandwidth is defined as BW = *f*_S_/(2∙OSR). Walden’s figure-of-merit (FOM_W_) is defined as

(10) $${\mathrm{FOM}}_{W}=\frac{\mathrm{Power}}{2\cdot \mathrm{ERBW}\cdot 2^{\mathrm{ENOB}}}\;[J/\mathrm{conv}.]$$

where effective resolution bandwidth (ERBW) is approximately half of the sampling frequency. The work [9] achieves a relatively good performance using the DAC mismatching error shaping. The SNR is increased from 69 to 97.9 dB using a relatively high OSR = 512; however, the opamp in the noise shaping filter consumes static power, leading to a relatively low FOM_W_. All the works except ours [12,18] use a multi-path comparator having an additional input pair for residue processing. The increased input-referred noise of the comparator can limit the achievable ADC performance [7]. The authors of [10,12,18] use 28, 40, and 14 nm CMOS processes and achieve a FOM_W_ better than ours; however, the power consumption of the SAR ADC usually decreases with the CMOS process scaling. Therefore, direct comparison is difficult. The DEM technique addresses the mismatch problem [9,19]. These works show slightly better FOM_S_ than ours, while our work achieves better FOM_W_. The work [19] uses the passive noise shaping filter; however, the comparator having three input branches increases the power and noise. Works [16,17,18,19,20] consume power > 100 μW, and it is difficult to use these works for the IoT demanding an ultra-low power.

Realized using the noise shaping filter with passive gain multiplication, the proposed ADC consumes the lowest power of 4.1 μW, leading to a favorable FOM_W_ of 11.8 fJ/conversion-step. Our work presents the effectiveness of the DEM using a behavioral model, which can further increase SNDR. The result shows that the proposed approach of noise shaping is promising for improving the performance of the SAR ADC. Although the proposed ADC achieves a moderate FoM_S_, low power consumption at a medium conversation rate is suitable for IoT. The FoM_S_ can be further enhanced by implementing a more advanced CMOS process. There are many application scenarios of the proposed ADC since sensing analog signals is necessary for various IoT systems. For the sensor interface in these applications, very low power consumption is required to provide a long battery life. Examples include various battery-operated sensing systems [28], deployed in various biomedical, home, industrial, and environment monitoring objects.

## 4. Conclusions

We propose a noise-shaping SAR ADC featuring a passive gain multiplication technique and successfully verify the approach using a chip fabricated in a 0.18 μm CMOS process. We embed the charge pump in the noise shaping filter to boost the gain without static power consumption, which effectively deals with the residue voltage attenuation. The proposed approach consists of a few capacitors and switches, allowing noise shaping implemented with low power and small area. We present the comparator with reduced kickback noise that effectively handles the tradeoff between noise suppression and chip area. The energy-efficient MCS technique is effectively combined with thermometer-coded CDAC, which reduces the settling error in the DAC. The effect of filter capacitor size and process-induced mismatch in the CDAC is investigated using a behavioral model of the ADC. Additionally, we propose a simple DEM implementation, confirmed using the behavioral simulations. The ADC is fabricated using a 0.18 μm CMOS process. Measured data show the successful operation of the proposed noise shaping technique. The ADC achieves measured SNDR of 66.5 dB and SFDR of 79.1 dB with FoM of 11.8 fJ/conversion-step. The main contribution of this paper is validating a simple and power-efficient noise shaping technique for the SAR ADC using the embedded passive gain multiplication. The proposed approach tackles the drawback of increased power and extra noise of the active noise shaping and limited noise suppression of the passive noise shaping. Future research direction will be implementing the SAR ADC using an advanced CMOS node to increase the bandwidth. Experimental validation of the proposed DEM is also demanded. The result will be useful for realizing a power-efficient SAR ADC for various IoT sensor systems.

## Figures and Tables

**Table 1:** List of noise transfer function.

	NTF	Noise Suppression
Ideal	1 − z^−1^	−10 dB
[13]	1 − 0.5z^−1^	−5.26 dB
[18]	(1 − 0.5z^−1^)/(1 + 0.5z^−1^)	−8.68 dB
Proposed	(1 − 0.79z^−1^)/(1 + 0.45z^−1^)	−12.49 dB

**Table 2:** Performance comparison.

	[9]	[10]	[12]	[15]	[16]	[17]	[18]	[19]	[20] *	This Work
Filter type	Active	Active	Active	Passive	Passive	Passive	Passive	Passive	Passive	Passive
OP-amp free	No	No	Yes	Yes	Yes	Yes	Yes	Yes	Yes	Yes
Filter order	1	3	2	1	2	2	1	2	1	1
Extra input for comparator (No.)	Yes (2)	Yes (2)	No	Yes (2)	Yes (2)	Yes (3)	No	Yes (3)	Yes (2)	No
CDAC (bit)	12	12	9	10	8	9	10	10	10	9
Supply (V)	1.2	1.55/0.75	1.1	1.2	1	1.1	0.9	1.0	1	1.8
Bandwidth (kHz)	1	2	625	125	8000	262	40,000	100	2000	3
OSR	512	25	8	8	4	16	4	16	25	20
Power (μW)	15.7	37.1	84	61	253	143	1250	118	561	4.1
Process (nm)	55	28	40	130	65	40	14	28	65	180
FoMS (dB)	180	175	178	167	169	173	171.7	173	176.8	170
FoMW (fJ/conv.-step)	85	5	9	7	10.9	33	8.9	251	16	11.8

* Simulation results.
